# Risk Factors for Colorectal Cancer in Inpatients With Ulcerative Colitis: A Nationwide Cross-Sectional Analysis

**DOI:** 10.7759/cureus.27114

**Published:** 2022-07-21

**Authors:** Sravani Kommuru, Syed Nurul Aziz, Sowmya Sagireddy, Gagan Kaur, Satya Rijal, Chia Chi Loh, Yakub Ibrahim, Viralkumar Patel

**Affiliations:** 1 Internal Medicine, Dr. Pinnamaneni Siddhartha Institute of Medical Sciences And Research Foundation, Vijayawada, IND; 2 Internal Medicine, Shaheed Suhrawardy Medical College, Dhaka, BGD; 3 Internal Medicine, Coney Island Hospital, New York City, USA; 4 Medicine and Surgery, Sri Guru Ram Das Institute of Medical Sciences and Research, Amritsar, IND; 5 Medicine and Surgery, Southeast University Medical College, Nanjing, CHN; 6 Internal Medicine, Manipal University College Malaysia, Melaka, MYS; 7 Emergency Medicine, Southend University Hospital, Essex, GBR; 8 Internal Medicine, Sarasota Memorial Health Care System, Sarasota, USA

**Keywords:** risk-factors, risk factors, acquired immune deficiency syndrome (aids), colorectal cancer, colorectal cancer, ulcerative colitis (uc)

## Abstract

Objectives

The objective is to study the demographic and geographical factors that increase the risk of colorectal cancer (CRC) in inpatients with ulcerative colitis (UC) and evaluate the mortality risk and hospitalization outcomes in terms of length of stay (LOS) and cost of care in patients with CRC in UC.

Methods

We conducted a cross-sectional study using the nationwide inpatient sample (NIS, 2019). We included 78,835 inpatients (age 15-65 years) hospitalized on emergency-based admissions with a primary diagnosis of UC. The study sample was divided by the presence of CRC. Categorical and continuous data were analyzed using Pearson’s chi-square test and independent-sample t-test respectively. Independent binomial logistic regression models were used to evaluate the odds ratio (OR) of predictors associated with CRC in patients with UC compared to non-CRC.

Results

The prevalence of CRC in inpatients with UC was 0.2%, and the mean age for admission of patients with UC with CRC was 49.6 years (SD ± 10.29). A directly proportionate relationship exists between increasing age and the risk of CRC in UC inpatients with 10 times higher odds seen in 51-65 years of age (OR 10.0, 95% CI 5.11-19.61). Males (OR 2.15, 95% CI 1.49-3.08) and Hispanics (OR 1.69, 95% CI 1.04-2.74) are at higher odds for CRC compared to their counterparts. Acquired immunodeficiency syndrome (AIDS) was associated with increased odds (OR 6.23, 95% CI 2.48-15.68) for CRC in UC inpatients. There existed an increased association for CRC in UC inpatients with complicated hypertension, and alcohol and drug abuse but was statistically non-significant. As per the adjusted regression model, CRC in UC inpatients increased the risk of in-hospital mortality (OR 41.09, 95% CI 19.49-86.58).

Conclusions

CRC was more prevalent in middle-aged Caucasian males with UC and those with chronic comorbidities including complicated diabetes and hypertension, alcohol abuse, and AIDS. Patients with UC and AIDS were found to have greater odds of developing CRC. A high index of clinical suspicion is needed in the management of these patient groups as the inpatient mortality risk was higher in UC inpatients with CRC.

## Introduction

Colorectal cancer (CRC) is the second leading cause of cancer-related death in the United States (US) [1]. According to the national cancer institute, the age-adjusted new case rate for CRC between the year 2015 and 2019 was 37.7 per 100,000 men and women per year, and the death rate was 13.4 per 100,00 men and women per year [2]. The median age of diagnosis is 66 years old, and males are more affected than females [2]. It is known that the risk of mortality from CRC increases with age. The highest mortality due to CRC is seen between 65 and 74 years of age; however, the age-adjusted mortality rates have been falling by 2% each year from 2010 to 2019. The risk of CRC is nearly doubled in patients with inflammatory bowel disease (IBD) compared to the general population [3].

Ulcerative colitis (UC) is a chronic IBD caused by various immunologic changes to the large intestine. The risk of CRC is higher in patients with a long duration of UC [4]. UC-CRC accounts for nearly one percent of all CRC cases. Some risk factors contributing to UC-related CRC are duration and extent of disease and co-diagnosis of primary sclerosing cholangitis (PSC) [5]. More than 1,300 CRC cases and 600 deaths from CRC were detected in a two-nation cohort study that included more than 95,000 individuals with UC. There was a 60% greater risk of a diagnosis of CRC and death when compared to matched reference people from the general population [3].

Chromosomal instability (CIN) and microsatellite instability lead to mucosal dysplasia via chronic inflammation, a major contributing factor to carcinogenesis, especially in UC [4]. Many factors contribute to the development of CRC, such as age, smoking, alcohol consumption, intake of red and processed meats, medical conditions such as diabetes mellitus, history of IBD, and obesity [4].

Despite these risk factors, 75% of cases occur without any predisposition [5]. Screening programs have played a pivotal role in early detection and management. CRC incidence is noted to be rising in all ethnic groups, found to be most prevalent in African Americans and Native Americans than in Caucasians [6]. Patients with UC-CRC are known to have a worse prognosis than CRC patients without UC [4]. In this study, we aim to study demographic characteristics and comorbidities that increase the risk of CRC in patients with UC. Further, we will evaluate the mortality risk and hospitalization outcomes including length of stay (LOS) and cost of care in patients with CRC in UC.

## Materials and methods

Study data

We conducted a cross-sectional study using the nationwide inpatient sample (NIS, 2019). The NIS is the largest hospital-based publicly available de-identified data that covers non-federal community hospitals from 48 states in the US. According to the agency for healthcare research and quality (AHRQ) and the department of health and human services, the utilization of the NIS does not require approval from an institutional review board [7].

Inclusion and exclusion criteria

We included 78,835 inpatients (age 15-65 years) hospitalized on emergency-based admissions with a primary diagnosis of UC. The study sample was divided by the presence of CRC: non-CRC (N = 78,685) versus CRC (N = 150) cohorts. We excluded the inpatients for other primary diagnoses as our study population was only focused on those patients primarily managed for UC.

Variables

The variable of interest included demographic characteristics (age, sex, and race), and comorbidities which are the co-diagnoses in the patient records, and we included acquired immunodeficiency syndrome (AIDS), diabetes with complications, hypertension (complicated), chronic pulmonary disease, alcohol abuse, depression, and drug abuse. The hospitalization outcomes of interest included: severity of illness which was measured using the all-patient refined drugs (APR-DRGs) in the NIS, and the LOS, total charges, and in-hospital mortality (all-cause) [7].

Statistical analysis

We used Pearson’s chi-square test and descriptive statistics for categorical data and an independent-sample t-test for continuous data (LOS and total charges) to measure the differences between UC inpatients with non-CRC vs. CRC. Independent binomial logistic regression models were used to evaluate the odds ratio (OR) of predictors associated with CRC in patients with UC compared to non-CRC (as a reference category); another regression model was conducted to evaluate the risk factors of all-cause in-hospital mortality in UC inpatients. A P-value <0.05 was used to determine the statistical significance test result and all analyses were conducted using the statistical package for the social sciences (SPSS) version 27 (IBM Corp., Armonk, NY).

## Results

Our study inpatients were majorly constituted of middle-aged adults (31-50 years, 42.8%), females (52.5%), and Caucasian (70.9%). The prevalence of CRC in inpatients with UC was 0.2% (N = 150). The mean age of the CRC cohort was 49.6 years and about 43% were middle-aged males (70%) and Caucasians (75.9%). There existed a statistically significant difference between CRC and non-CRC cohorts in terms of age, sex, and race/ethnicity. Comorbid AIDS (3.3% vs. 0.4%), diabetes with complications (6.7% vs. 3.5%), complicated hypertension (6.7% vs. 3%), and alcohol abuse (6.7% vs. 2.2%) were significantly prevalent in the UC inpatient with CRC. There existed a significant difference in the severity of illness between cohorts (P < 0.001) with a higher proportion of UC with CRC inpatients having moderate (63.3%) and major (26.7%) loss of body functioning. The in-hospital mortality rate was higher in the CRC cohort (6.7% vs. 0.1%, P < 0.001). Also, there existed a statistically significant higher difference in mean LOS and mean total charges in UC inpatients with CRC compared to the non-CRC cohort as shown in Table 1.

**Table 1:** Differences in demographics and hospital outcomes in ulcerative colitis inpatients CRC: colorectal cancer; AIDS: acquired immunodeficiency syndrome; LOS: length of stay; SD: standard deviation

Variable	CRC (no)	CRC (yes)	Total	P-value
Number of inpatients	78685	150	78835	-
Mean age at admission, in years	39.2 (SD ± 13.83)	49.6 (SD ± 10.29)	-	<0.001
Age at admission, in %				
15-30 years	31.7	6.7	31.7	<0.001
31-50 years	42.8	43.3	42.8	
51-65 years	25.4	50.0	25.5	
Sex, in %				
Male	47.4	70.0	47.5	<0.001
Female	52.6	30.0	52.5	
Race/ethnicity, in %				
Caucasian	70.9	75.9	70.9	<0.001
African American	15.6	3.4	15.6	
Hispanic	8.6	13.8	8.6	
Asian/Native American/Other	4.9	6.9	4.9	
Comorbidities, in %				
AIDS	0.4	3.3	0.4	<0.001
Diabetes with complications	3.5	6.7	3.5	0.035
Hypertension, complicated	3.0	6.7	3.0	0.010
Chronic pulmonary disease	12.1	10.0	12.1	0.425
Alcohol abuse	2.2	6.7	2.2	<0.001
Depression	14.7	6.7	14.7	0.005
Drug abuse	4.8	6.7	4.8	0.281
Severity of illness, in %				
Minor loss of function	33.1	10.0	33.0	<0.001
Moderate loss of function	48.4	63.3	48.5	
Major loss of function	18.5	26.7	18.5	
Other outcomes				
Mean LOS, in days	4.8 (SD ± 5.16)	10.7 (SD ± 19.62)	-	<0.001
Mean total charges, in $	51,294	158,984	-	<0.001
In-hospital mortality, in %	0.1	6.7	0.1	<0.001

There existed a proportionate relationship between increasing age and the risk of CRC in UC inpatients with ten times higher odds seen in 51-65 years of age (OR 10.0, 95% CI 5.11-19.61). Males (OR 2.15, 95% CI 1.49-3.08) and Hispanics (OR 1.69, 95% CI 1.04-2.74) are at higher odds for CRC compared to their counterparts. UC inpatients with CRC had four times higher odds for moderate-to-major loss of body functioning. Among the comorbidities, only AIDS was associated with increased odds (OR 6.23, 95% CI 2.48-15.68) for CRC in UC inpatients. There existed an increased association for CRC in UC inpatients with complicated hypertension, and alcohol and drug abuse but was statistically non-significant as shown in Table 2.

**Table 2:** Risk factors for colorectal cancer in ulcerative colitis inpatients AIDS: acquired immunodeficiency syndrome

Variable	Odds ratio	95% Confidence interval		P-value
		Lower limit	Upper limit	
Age at admission				
15-30 years	Reference			
31-50 years	5.00	2.55	9.80	<0.001
51-65 years	10.01	5.11	19.61	<0.001
Sex				
Female	Reference			
Male	2.15	1.49	3.08	<0.001
Race/ethnicity				
Caucasian	Reference			
African American	0.19	0.08	0.47	<0.001
Hispanic	1.69	1.04	2.74	0.033
Asian/Native American/Other	1.48	0.77	2.84	0.238
Severity of illness				
Minor loss of function	Reference			
Moderate loss of function	3.87	2.24	6.70	<0.001
Major loss of function	4.13	2.26	7.55	<0.001
Comorbidities				
None	Reference			
AIDS	6.23	2.48	15.68	<0.001
Diabetes with complications	1.05	0.90	1.21	0.649
Hypertension, complicated	1.40	0.71	2.79	0.335
Chronic pulmonary disease	0.79	0.46	1.38	0.412
Alcohol abuse	1.83	0.94	3.58	0.078
Depression	0.39	0.21	0.76	0.005
Drug abuse	1.24	0.64	2.41	0.528

Among the risk factors that increased the likelihood of in-hospital mortality in UC inpatients were 51-65 years of age (OR 7.48, 95% CI 3.78-14.78), and comorbid complicate hypertension (OR 4.94, 95% CI 2.93-8.31). Sex and race/ethnicity and other comorbidities were not significantly associated with increased in-hospital mortality risk. As per the adjusted regression model, CRC in UC inpatients increased the risk of in-hospital mortality (OR 41.09, 95% CI 19.49-86.58) as shown in Table 3.

**Table 3:** Risk factors for in-hospital mortality in ulcerative colitis inpatients AIDS: acquired immunodeficiency syndrome

Variable	Odds ratio	95% Confidence interval		P-value
		Lower limit	Upper limit	
Age at admission				
15-30 years	Reference			
31-50 years	<0.001	<0.001	-	0.950
51-65 years	7.48	3.78	14.78	<0.001
Sex				
Female	Reference			
Male	1.09	0.72	1.68	0.665
Race/ethnicity				
Caucasian	Reference			
African American	0.61	0.31	1.23	0.167
Hispanic	0.68	0.27	1.71	0.408
Asian/Native American/Other	1.32	0.53	3.29	0.553
Comorbidities				
None	Reference			
AIDS	<0.001	<0.001	-	0.994
Diabetes with complications	0.74	0.35	1.56	0.429
Hypertension, complicated	4.94	2.93	8.31	<0.001
Chronic pulmonary disease	1.63	1.01	2.63	0.045
Alcohol abuse	1.74	0.68	4.41	0.246
Depression	0.59	0.31	1.17	0.135
Drug abuse	0.95	0.37	2.46	0.911
Colorectal cancer	41.09	19.49	86.58	<0.001

## Discussion

The magnitude of risk for developing CRC in patients with UC is largely debated and the existing data is conflicting and heterogeneous. A study conducted by Dyson et al. found that the cumulative risk of developing CRC in UC patients is 2% to 10% over a period of ten years [8]. On the other hand, a meta-analysis stated that the risk of CRC was increased in IBD but was not as high as reported in earlier studies; the pooled standardized incidence rate in their study was 1.7 [9]. Along similar lines, we found that the prevalence of CRC in our study population was relatively low (0.2%), but we also found that it is one of the leading causes of mortality in patients with UC. In line with our findings, previous studies have also indicated that CRC is one of the leading causes of mortality and morbidity in patients with UC and accounted for nearly 10% to 15% of deaths [10].

Patients with long-term UC are at an increased risk of developing CRC [8]. This increased risk of CRC can be attributed to both genetic and acquired causes [8]. Confirmed risk factors for colitis-associated CRC include duration and severity of disease, the extent of colitis, presence of coexisting primary sclerosing cholangitis, and a family history of CRC. Patients with left-sided colitis carry moderate risk and those with pan-colitis are at the highest risk of CRC [8]. The adenoma-carcinoma sequence, which is associated with the sporadic form of colon cancer, has a role in the development of UC-CRC. The major genetic derangements in CRC are CIN and microsatellite instability (MSI). Studies have shown that these derangements occurred in the same frequency (81% CIN and 15% MSI), but the timing and sequence were different [11]. Sporadic CRC usually occurs towards the end of the adenoma-carcinoma sequence; however, in UC-CRC the sequence of mutations is different, and CRC develops at an earlier stage. Chronic inflammation can also lead to CRC in UC patients. Studies have also implicated that cyclooxygenase-2 messenger (COX-2), ribonucleic acid (RNA), and COX-2 protein overexpression which led to chronic inflammation was also associated with early-onset CRC in UC patients [4,11]. To conclude there has been evidence in the literature that suggests controlling the inflammation and mucosal damage in the cases of UC-CRC is crucial [4].

The mean age for developing CRC is relatively lower in patients with UC compared to patients with sporadic CRC [8]. We observed, that CRC was much more prevalent in people between 51 and 65 years. Aniwan et al. conducted a population-based cohort study which stated that the overall mortality rate in UC was lower in patients between 20 and 60 years of age [12]. This study also found that the risk of mortality in women with UC is similar to that of the general population in the US [12]. Along similar lines, we found that there was a statistically significant increase in mortality among the older age group (51-65 years). Our study has shown that males are at two times increased risk of mortality compared to females. In addition, we found that UC-associated CRC was much more prevalent among Caucasians, but Hispanics had about two times higher odds of developing CRC. Despite differences in prevalence rates among different ethnicities, we found that there is no statistically significant difference in mortality among these groups.

Association between AIDS and CRC has not been explored in detail, but our study demonstrated that UC patients with AIDS were six times more likely to develop CRC. After the introduction of antiretroviral therapy, the prevalence of AIDS has increased with improved survival [13]. Patients with AIDS over 50 years have been known to be at greater risk and screening is highly recommended in this group [14]. A study done by Bini, et al. pointed the duration of AIDS and cluster of differentiation 4 (CD4) cell count as independent risk predictors of distal colonic lesions in CRC [15]. Previous studies have pressed upon timely and adequate CRC screenings in the HIV population [13].

Treatment outcomes in patients with UC-associated CRC have been studied in terms of survival rates, local recurrences distant metastases, and other factors like postoperative complications. Studies have shown that UC-CRC patients required a more radical surgery (total proctocolectomy) [16]. Patients with UC-CRC were found to have rare and aggressive histological subtypes of CRC which led to increased risk for metastasis, but in a study conducted by Vitali et al. it was stated that there existed no statistically significant difference in overall survival rates and disease-free survival rates between sporadic and UC associated CRC [17]. In our study, we found that the in-hospital mortality rate was higher by 41 times in the UC-CRC group compared to the overall UC population. We also found that patients with UC-associated CRC had a moderate to major loss of function, increased mean LOS and increased mean total cost. Surgical site infections are known to be one of the common complications after a colorectal surgeries leading to longer hospital stay and increased morbidity [18]. The cost of care upon discharge is often increased in such pathologies due to increased need of elective and urgent procedures [18]. This could explain the mean cost of care in UC-CRC group being three times greater than UC with no CRC in our study population.

Our study report should be considered with some limitations. Given the nature of the cross-sectional retrospective analysis, we are unable to establish a causal relationship. We also acknowledge that there may be underreporting of comorbidities as this dataset lacks patient-level clinical information and the diagnoses are grouped by diagnostic codes. However, NIS offers a population-based perspective hence findings of our study can be generalized. Information is coded independently by practitioners hence there is minimal reporting bias. This is a large dataset also gives us the opportunity to work on a bigger sample and hence there is greater power in the study.

## Conclusions

CRC was more prevalent in middle-aged Caucasian males with UC. The risk of CRC in UC inpatients increases with age, and males and Hispanics have a higher likelihood of developing CRC among patients with UC. These at-risk patients’ have a higher risk of moderate-to-major loss of body function, and the in-hospital mortality risk was increased by 41 times in UC patients with CRC. Among the analyzed comorbidities, patients with AIDS were found to have greater odds of developing CRC than patients with UC. A high index of clinical suspicion is needed in the management of these patient groups.
